# *De novo* designed proteins neutralize lethal snake venom toxins

**DOI:** 10.21203/rs.3.rs-4402792/v1

**Published:** 2024-05-17

**Authors:** Susana Vázquez Torres, Melisa Benard Valle, Stephen P. Mackessy, Stefanie K. Menzies, Nicholas R. Casewell, Shirin Ahmadi, Nick J. Burlet, Edin Muratspahić, Isaac Sappington, Max D. Overath, Esperanza Rivera-de-Torre, Jann Ledergerber, Andreas H. Laustsen, Kim Boddum, Asim K. Bera, Alex Kang, Evans Brackenbrough, Iara A. Cardoso, Edouard P. Crittenden, Rebecca J. Edge, Justin Decarreau, Robert J. Ragotte, Arvind S. Pillai, Mohamad Abedi, Hannah L. Han, Stacey R. Gerben, Analisa Murray, Rebecca Skotheim, Lynda Stuart, Lance Stewart, Thomas J. A. Fryer, Timothy P. Jenkins, David Baker

**Affiliations:** 1. Department of Biochemistry, University of Washington, Seattle, WA, USA.; 2. Institute for Protein Design, University of Washington, Seattle, WA, USA.; 3. Graduate Program in Biological Physics, Structure and Design, University of Washington, Seattle, WA 98105, USA.; 4. Department of Biotechnology and Biomedicine, Technical University of Denmark, Kongens Lyngby, Denmark.; 5. Department of Biological Sciences, University of Northern Colorado, Greeley, CO, 80639, USA.; 6. Centre for Snakebite Research & Interventions, Liverpool School of Tropical Medicine, Pembroke Place, Liverpool L3 5QA, UK.; 7. Centre for Drugs & Diagnostics, Liverpool School of Tropical Medicine, Pembroke Place, Liverpool L3 5QA, UK.; 8. Biomedical & Life Sciences, Faculty of Health and Medicine, Lancaster University, Lancaster, United Kingdom LA1 4YG8.; 9. Sophion Bioscience, DK-2750 Ballerup, Denmark; 10. Department of Infection Biology and Microbiomes, Institute of Infection, Veterinary and Ecological Sciences, University of Liverpool, Liverpool, L3 5RF, United Kingdom.; 11. Media Lab, Massachusetts Institute of Technology, 77 Massachusetts Avenue, Cambridge, 02139, MA, USA; 12. Howard Hughes Medical Institute, University of Washington, Seattle, WA 98105,USA.

## Abstract

Snakebite envenoming remains a devastating and neglected tropical disease, claiming over 100,000 lives annually and causing severe complications and long-lasting disabilities for many more^1,2^. Three-finger toxins (3FTx) are highly toxic components of elapid snake venoms that can cause diverse pathologies, including severe tissue damage^3^ and inhibition of nicotinic acetylcholine receptors (nAChRs) resulting in life-threatening neurotoxicity^4^. Currently, the only available treatments for snakebite consist of polyclonal antibodies derived from the plasma of immunized animals, which have high cost and limited efficacy against 3FTxs^5,6,7^. Here, we use deep learning methods to *de novo* design proteins to bind short- and long-chain α-neurotoxins and cytotoxins from the 3FTx family. With limited experimental screening, we obtain protein designs with remarkable thermal stability, high binding affinity, and near-atomic level agreement with the computational models. The designed proteins effectively neutralize all three 3FTx sub-families *in vitro* and protect mice from a lethal neurotoxin challenge. Such potent, stable, and readily manufacturable toxin-neutralizing proteins could provide the basis for safer, cost-effective, and widely accessible next-generation antivenom therapeutics. Beyond snakebite, our computational design methodology should help democratize therapeutic discovery, particularly in resource-limited settings, by substantially reducing costs and resource requirements for development of therapies to neglected tropical diseases.

Snakebite envenoming represents a public health threat in many developing regions, notably impacting on low resource settings in sub-Saharan Africa, South Asia, Papua New Guinea, and Latin America^2^. With over two million annual cases, snakebite results in 100,000 fatalities and 300,000 permanent disabilities^1^. In 2017, the World Health Organization (WHO) listed snakebite envenoming as a highest priority neglected tropical disease^8^. Nonetheless, limited resources have been dedicated to improving current antivenom treatments^2^. These therapies rely on plasma-derived polyclonal antibodies from hyperimmunized animals, complemented by medical and surgical care^9^. While instrumental in saving lives, antivenom accessibility is hindered by high production costs and inadequate cold chain infrastructure in remote areas^9^. Serious adverse effects, including hypersensitivity (such as anaphylaxis) and pyrogenic reactions represent additional challenges during antivenom administration^2,10,11^. Furthermore, these treatments are often ineffective in counteracting neurotoxicity and tissue necrosis due to suboptimal concentrations of neutralizing antibodies against three-finger toxins (3FTxs)^5,6,7^. This inefficacy stems from the limited immunogenicity of 3FTxs in the antivenom production animal, resulting in a failure to elicit a strong antibody response^12^. Additional issues arise due to delayed administration of antivenom treatment^13^. Antibody^14,15,16,17,18,19,20,21,22^ and non-antibody-based therapeutics^23,24,25,26,27,28,29,30^ have been tested in preclinical studies, but the development of these types of molecules require either immunization of animals or development of large libraries that require extensive selection, screening, and optimization efforts^31^.

We reasoned that *de novo* design approaches could have advantages over traditional methods of antivenom development. First, *de novo* protein design does not rely on animal immunization, and yields proteins that can be manufactured using recombinant DNA technology, thereby creating a source for continuous production of product with limited batch-to-batch variation. Second, computational design enables the creation of binding proteins with high affinity and specificity without needing extensive experimental screening programs that often rely on pure toxins, which can be challenging to isolate from whole venoms or generate via recombinant expression^32,33^. Third, the small size of designed proteins could offer enhanced tissue penetration^34^ compared to large antibodies, enabling rapid toxin neutralization and thereby potentially be particularly effective in neutralizing non-systemic toxicities, such as those leading to local tissue damage. Fourth, designed proteins can have high thermal stability^35^ and can be produced using low-cost microbial fermentation strategies, which could help enable the development and deployment of new antivenom therapeutics at reduced cost^36^. Hence, we set out to use the deep learning based RFdiffusion method^35^ to design antivenoms for short- and long-chain α-neurotoxins and cytotoxins from the 3FTx snake venom toxin family.

## Design of α-neurotoxin binding proteins

**Figure 1.**
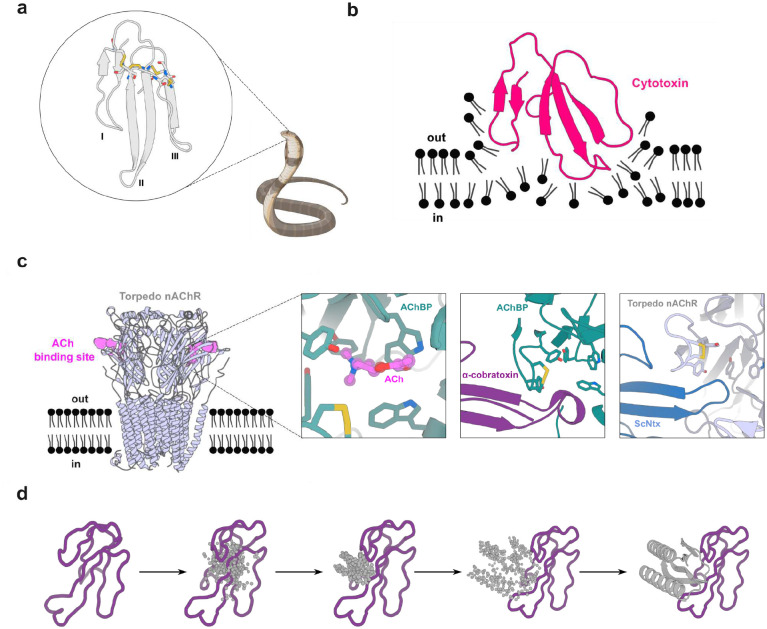
Targets of 3 finger snake toxins (3FTxs). **(a)** Structure of 3FTxs^60^ (PDB ID: 1QKD). Highly conserved cysteine residues are highlighted in sticks and each of the three fingers indicated (I-III). **(b)** Representation of a type IA cytotoxin^61^ (dark pink) (PDB ID: 5NQ4) interacting with a lipid bilayer. **(c)** Muscle acetylcholine (Torpedo) receptor (light blue) (PDB ID: 7Z14)^39^. Acetylcholine (ACh) binding site is depicted in violet. Left inset: Close-up of the acetylcholine binding protein (AChBP) (teal) (PDB ID: 3WIP) bound to Ach^62^ (violet). A set of aromatic residues form a cage around the neurotransmitter. Middle: Close-up of α-cobratoxin (dark purple) blocking access to the ACh binding site in AChBP (teal) (PDB ID: 1YI5)^63^. Right: Close-up of ScNtx (dark blue) blocking access to the ACh binding site in the Torpedo receptor (light blue) (PDB ID: 7Z14)^39^. **(d)** Schematic showing α-cobratoxin binder design using RFdiffusion. Starting from a random distribution of residues around the specified β-strands in the target toxin (dark purple), successive RFdiffusion denoising steps progressively remove the noise leading at the end of the trajectory to a folded structure interacting with α-cobratoxin β-strands. Panels **(a)**, **(b)**, and **(c)** were created with BioRender.com

α-neurotoxins, a prominent subclass of 3FTxs, adopt a multi-stranded β-structure with three extended loops protruding from a hydrophobic compact core stabilized by highly-conserved disulfide bridges^37,38^ (Figure 1a). Short-chain and long-chain α-neurotoxins differ in length and number of disulfide bonds. Despite sequence homology, α-neurotoxins display distinct pharmacological profiles across nAChR subtypes: short- and long-chain α-neurotoxins inhibit muscle-type nAChRs, but only long-chain α-neurotoxins strongly bind to neuronal α7 nAChRs^39^ (Figure 1c). As many elapid snake species possess venoms that derive their lethal effect from these toxins, it is crucial to neutralize both types of α-neurotoxins to achieve therapeutic efficacy and prevent venom-induced lethality in victims envenomed by these snakes.

We chose to target our design efforts against the neurotoxin edge β-strands (previously discovered monoclonal antibodies in contrast mimic the nAChR binding site^14,21,22^), focusing on binding modes blocking neurotoxin binding to nAChRs through steric hindrance. Secondary structure and block adjacency tensors were provided to the RFdiffusion model to specify desired β-strand interactions between the designed binder and target α-neurotoxins (see Methods). For each secondary structure tensor, interactions between one binder β-strand and a target neurotoxin β-strand were encoded in a block adjacency tensor, preconditioning RFdiffusion towards β-strand pairing complexes. Following backbone generation through RFdiffusion denoising trajectories, sequence design was carried out using ProteinMPNN, the resulting designs were filtered based on AF2 *initial guess*^40^ and Rosetta metrics, and the most promising candidates selected for experimental characterization.

**Figure 2.**
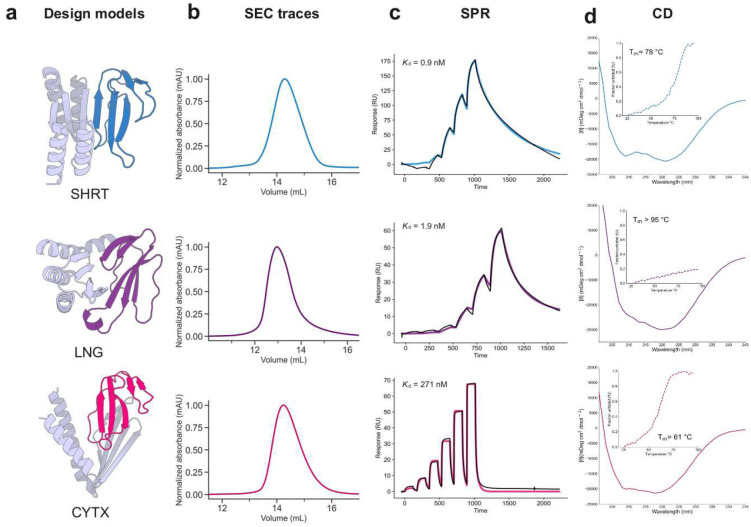
Experimental characterization of 3FTx binding proteins. **(a)** Design models of protein binders (gray) bound to their 3FTx targets (dark blue: ScNtx, dark purple: α-cobratoxin, dark pink: consensus cytotoxin). **(b)** SEC traces of purified proteins. **(c)** SPR binding affinity measurements. Colored solid lines represent fits using the heterogeneous ligand model, with dissociation constant (*K*_d_) values derived from these fits**. (d)** CD data confirms the presence of αβ-secondary structure in the 3FTx binding proteins and their thermal stability (inset).

**Figure 3.**
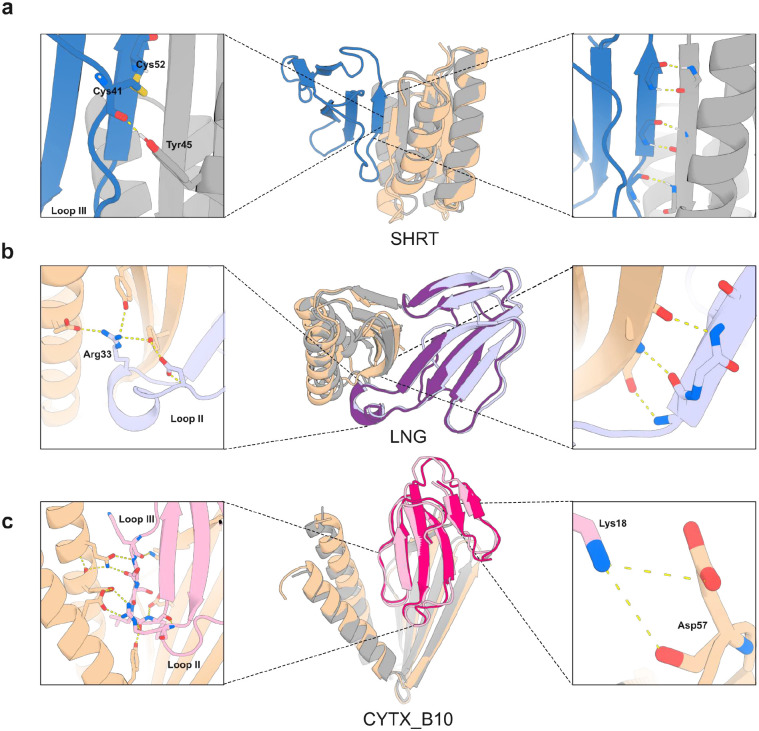
Crystal structures of 3FTx binding proteins closely match design models. **(a)** Apo-state crystal structure of SHRT design. Left: Hydrogen bonding between the carbonyl oxygen of Cys41 in ScNtx (dark blue) and the side chain of Tyr45 in the SHRT design model (gray). Middle: Overlay of SHRT design model (gray) with crystal structure (wheat). Right: Backbone hydrogen bonding between the SHRT design model (gray) and ScNtx (dark blue) β strands. **(b)** Crystal structure of LNG design in complex with α-cobratoxin. Left: Cross-interface hydrogen-bond network involving Arg33 at loop II in α-cobratoxin (light purple) and Glu69, Tyr40, and Tyr49 in LNG crystal structure (wheat). Middle: Overlay of LNG design model (gray) bound to α-cobratoxin (dark purple) with crystal structure of binder (wheat) bound to toxin (light purple). Right: Backbone hydrogen bonding between crystal structure of designed binder (wheat) and α-cobratoxin (light purple) β strands. **(c)** Crystal structure of CYTX_B10 design in complex with *Naja pallida* cytotoxin. Left: Cross-interface electrostatic interaction network between loops III and II of *Naja pallida* cytotoxin (light pink) and binder crystal structure (wheat). Middle: Overlay of CYTX_B10 design model (gray) bound to toxin (dark pink) with crystal structure of binder (wheat) bound to *Naja pallida* cytotoxin (light pink). Right: Salt bridge between positively charged Lys18 in cytotoxin (light pink) and Asp57 in the binder crystal structure (wheat).

We targeted short-chain α-neurotoxins using a previously designed consensus toxin derived from elapid snakes (ScNtx)^41^ as a representative template. Synthetic genes encoding 44 designs targeting ScNTx were screened via yeast surface display (YSD), and one candidate was identified to bind to ScNTx with a dissociation constant (*K*_d_) of 842 nM as confirmed by bio-layer interferometry (BLI) (Supplementary Figure S1). Partial diffusion optimization^42^ improved the binding affinity of the ScNTx binder (SHRT) to 0.9 nM, as determined by surface plasmon resonance (SPR) following the screening of 78 designs (Figure 2c, top row; a very similar value of 0.7 nM was obtained by BLI (Supplementary Figure S2)). The optimized binder displayed a single monomeric peak on size exclusion chromatography (SEC), characteristic αβ-protein circular dichroism (CD) spectra, and thermal stability with a melting temperature (T_m_) of 78 °C (Figure 2d, top row). Using X-ray crystallography, we determined the structure of the SHRT design in the apo state, which closely matched the computational design model (2.58 Å resolution; 1.04 Å RMSD) (Figure 3a).

As a representative long-chain α-neurotoxin, we chose α-cobratoxin from *Naja kaouthia*, one of the most extensively characterized toxins from the 3FTx family^43^ (Figure 1). From 42 RFdiffusion designs against α-cobratoxin, one candidate had a binding affinity of 1.3 μM using BLI (Supplementary Figure S3). We again used partial diffusion to optimize the binding interface, and of 38 protein designs tested, the highest affinity binder (LNG) had a *K*_d_ of 1.9 nM measured by SPR (Figure 2c, middle row; BLI yielded a value of 6.7 nM (Supplementary Figure S4)). CD melting experiments revealed very high thermal stability (T_m_ > 95°C; Figure 2d, middle row).

Using X-ray crystallography, we determined the structure of the α-cobratoxin binder in complex with the target, which closely matched the computational design model (2.68 Å resolution; 0.42 Å RMSD over design, 0.61 Å over toxin; there is a slight deviation in the positioning of the toxin relative to the binder). As in the design model, the binder interacts with the central loop II of the neurotoxin, which is crucial for interaction of the toxin with muscle-type and neuronal α7 nAChRs^44,45^. This interaction is primarily mediated by backbone hydrogen bonding between a β strand in the designed binder and a β strand in the toxin (Fig. 3b). An arginine residue at position 33, located at the tip of loop II of α-cobratoxin, makes extensive electrostatic interactions with the binder (Fig 3b, left inset).

## Design of cytotoxin binding proteins

Cytotoxins, a prominent functional group within the 3FTx family found in cobra venoms, exert cytotoxic effects and induce local tissue damage by destabilizing phospholipid membranes^46^ (Figure 1b). Neutralizing these toxins is crucial to prevent severe sequelae, such as limb deformity, amputation, and lasting disabilities in snakebite victims^47^.

For targeting cytotoxins, we hypothesized that relying solely on β-strand pairing interactions might not adequately prevent cytotoxin insertion into membranes due to the critical role of their three-finger loops in membrane interaction and disruption^48,49,50,51^ (Figure 1d). Instead, we focused on binding directly to the cytotoxin three-finger loops by generating RFdiffusion-based protein backbones with hotspot residues defined within these regions (Figure 2a, bottom row). To increase the breadth of neutralization, we targeted a consensus sequence derived from 86 different snake cytotoxins (Type IA cytotoxin sub-subfamily; see Methods). Following ProteinMPNN and AF2 screening, partial diffusion was used to further optimize designs with the best metrics. A total of 55 protein designs were recombinantly expressed using *Escherichia coli*, and following SEC purification, the 18 designs with monomeric populations were tested in a luminescent cell viability assay. Of these, one protein binder (CYTX) had high solubility, with a single monomeric peak in SEC and had high neutralization activity against *Naja pallida* and *Naja nigricollis* whole venoms, known for their high cytotoxin content^52^ (Supplementary Figure S5). The *K*_d_ for the cytotoxin from *Naja pallida* was determined to be 271 nM via SPR (Figure 2c, bottom row). CYTX exhibited characteristic αβ-protein CD spectrum and was thermostable, with a T_m_ of 61°C (Figure 2d, bottom row).

Few designed binders have targeted loops, and hence we sought to solve the crystal structure of CYTX in complex with *Naja pallida* cytotoxin (Fig 3c). To reduce flexibility to favor crystallization, a disulfide bond was introduced within a flexible loop connecting the β-sheet segment to the two α-helices of CYTX, yielding a candidate (CYTX_B10) with improved thermal stability (T_m_= 70.3°C) and monomeric profile during SEC, but a slightly weaker *K*_d_ of 740 nM for *Naja pallida* cytotoxin (Supplementary Fig.S6). The structure of CYTX_B10 in complex with the target closely matched the computational design model (resolution: 2.0 Å; RMSD: 1.32 Å over design, 0.58 Å over toxin), revealing extensive electrostatic interactions involving side chain–main chain hydrogen bonds between cytotoxin loops II and III and the CYTX_B10 binder (Figure 3c, left inset). The unusual open fold of CYTX_B10 highlights the power of RFdiffusion to custom generate scaffolds shape-matched with protein targets, and the power of proteinMPNN to stabilize structures which violate common rules of protein structure (in this case lacking a central hydrophobic core).

## *In vitro* neutralization

**Figure 4.**
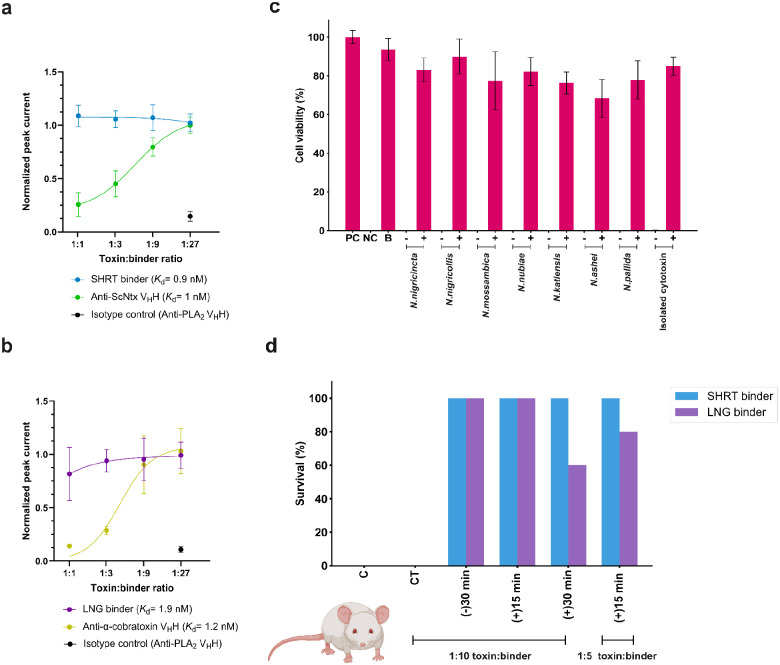
*In vitro* and *in vivo* efficacy of designed proteins against snake venom toxins. **(a)** Concentration-response curves comparing SHRT binder and anti-ScNtx V_H_H efficacy in preventing nAChR blocking by 1 IC_80_ of ScNtx. Data represent the toxin’s inhibition of ACh response, normalized to full ACh response, averaged within each group (*n*=16). **(b)** Concentration-response curves comparing the efficacy of LNG binder and anti-α-cobratoxin V_H_H in preventing nAChR blocking by 1 IC_80_ of α-cobratoxin. **(c)** Neutralization of the cytolytic effects of whole venoms from seven different *Naja* (*N*.) species and isolated cytotoxin by the CYTX binder. 2 IC_50_ of the whole venoms or toxin were pre-incubated with CYTX at a 1:5 molar ratio (toxin:binder). Keratinocyte media was used as a positive control (PC). Triton X-100 was used as a negative control (NC). CYTX binder (B) was used as a positive control. (−) denotes 2 IC_50_ of the whole venoms without binder, and (+) denotes venoms incubated with binder. Experiments were performed in triplicates, and results are expressed as mean ± SD. **(d)** Mice survival following lethal neurotoxin challenge (*n*=5). 3 LD_50_s of ScNtx or α-cobratoxin were preincubated for 30 minutes (−30 min) with the corresponding protein binders at 1:10 ratios and then administered IP into groups of five mice. Toxins administered IP following IP administration of binders at 1:10 or 1:5 molar ratios (toxin:binder) either after 15 (+15 min) or 30 minutes (+30 min) post-toxin injection. Controls included mice receiving toxins alone (C). Specificity was assessed via cross-treatment (CT) experiments, where non-target binders were preincubated with 3 LD_50_s of ScNtx or α-cobratoxin and administered IP. Signs of toxicity were observed, and deaths were recorded for a period of 24 hours. **(d)** was created with BioRender.com.

We assessed the ability of the designs to functionally neutralize α-neurotoxins in patch-clamp experiments using a human-derived rhabdomyosarcoma cell line expressing muscle-type nAChRs. When preincubated with ScNtx, the SHRT design achieved complete neutralization at a 1:1 molar ratio (toxin:binder), better than a previously characterized ScNtx nanobody (TPL1163_02_A01)^53^ (Figure 4a; a control nanobody targeting phospholipase A_2_ (PLA_2_) had no effect). Similarly, the LNG design had better neutralizing efficacy than a previously characterized α-cobratoxin nanobody (TPL1158_01_C09)^53^, achieving full protection at a 1:1 molar ratio (toxin:binder) (Figure 4b).

We used a cytotoxicity assay to evaluate the cross-reactivity of the CYTX design against various cobra whole venoms. Immortalized human keratinocytes (N/TERTs) were exposed to venoms from seven different *Naja* (*N*.) species, which prior proteomic analyses suggest consist primarily (~70%) of cytotoxins^54^. Pre-incubating CYTX with venoms (2 IC_50_s) at a 1:5 molar ratio (toxin:binder) provided 70–90% protection against venom-induced cytotoxicity (Figure 4c). Similarly, pre-incubation of the cytotoxin binder with isolated cytotoxin from *N. pallida* (2 IC_50_s) at a 1:5 molar ratio (toxin:binder) gave 85% protection against cytotoxicity (Figure 4c). However, preliminary studies indicated that the CYTX design, in 1:1, 1:2.5, and 1:5 molar ratios (toxin:binder), did not significantly decrease the size of the dermonecrotic lesions induced by intradermal *N. nigricollis* venom administration in a murine model^55^ (Supplementary Fig.S7); the affinity of CYTX likely needs to be further optimized for full *in vivo* neutralization of cytotoxins.

## *In vivo* protection

Given the encouraging *in vitro* neutralization for our anti-neurotoxin designs, we proceeded to *in vivo* studies. We determined the mean lethal dose (LD_50_) values for α-neurotoxins in male non-Swiss albino (NSA) mice via intraperitoneal (IP) administration; α-cobratoxin had an LD_50_ of 0.098 μg/g and ScNtx had an LD_50_ of 0.087 μg/g, in agreement with prior intravenous LD_50_s doses of these toxins (0.1 μg/g)^56^. We evaluated the *in vivo* neutralizing capacity of our neurotoxin-targeting protein binders by assessing survival over a 24-hour period following lethal neurotoxin challenge (3 LD_50_s) (Figure 4d). The SHRT binder provided complete protection (100%) to mice when pre-incubated and administered intraperitoneally with the corresponding short-chain neurotoxin at a 1:10 molar ratio of toxin to binder, but as expected did not neutralize the non-target α-cobratoxin. The LNG binder exhibited comparable efficacy, completely neutralizing α-cobratoxin but not the non-target ScNtx (Figure 4d, left). In rescue assays better mimicking a real-life snakebite scenario, complete protection (100%) was achieved when short-chain or long-chain α-neurotoxin binders were administered intraperitoneally at a 1:10 molar ratio (toxin:binder) 15 minutes after a lethal α-neurotoxin challenge (3 LD_50_s) (Figure 4d, middle). Administering the SHRT binder 30 minutes post-toxin injection also provided 100% protection against ScNtx, while the LNG binder conferred 60% protection against α-cobratoxin (Figure 4d, right). All surviving mice showed no evidence of limb or respiratory paralysis. At a 1:5 molar ratio (toxin:binder), IP administration of the SHRT design 15 minutes after toxin injection (3 LD_50_s) resulted in 100% survival, while the LNG binder provided 80% protection. Mice injected with the binder alone showed no negative effects at 24 and 48 hours post-injection, nor up to two weeks post-injection.

## DISCUSSION

Antivenoms based on animal-derived polyclonal antibodies have long been the cornerstone of snakebite envenoming therapy, but their application is hampered by their limited efficacy against toxins with low immunogenicity, their propensity to cause severe adverse reactions, and the inherent batch-to-batch variations and high production costs associated with their manufacture^57^. Thus, there has been a search for alternatives, with recombinant human monoclonal antibodies and nanobodies presenting a solution that can help overcome some of these limitations^58^. Our designed neurotoxin binders demonstrate comparable potency to the best immunoglobulin G antibodies and nanobodies reported in literature^58^, are highly stable and readily producible in microbial systems, and their small size (~100 amino acids) may possibly enable them to penetrate rapidly into deep tissue^34^. More generally, our *in silico* design approach avoids animal immunization and/or construction and multiple rounds of selection and/or screening of large libraries, providing a low-cost methodology for rapid development of toxin binders to the many components of snake venom when structural or sequence data exists for these targets. *De novo* designed proteins have high stability and are amenable to low cost manufacturing, which is key to effectively addressing snakebite envenoming as a neglected tropical disease. From the design perspective, the crystal structure of our cytotoxin binder highlights the ability of RFdiffusion to custom design scaffolds to match almost any target shape, and to generate binders to loop regions of proteins, with the inhibitory activity of the anti-cytotoxin designs directly supporting a role for the loops in membrane disruption.

Advancing the field to provide effective solutions for snakebite victims requires a collaborative effort involving the scientific community, the pharmaceutical industry, public health systems, and governments^2^. While traditional antivenoms will likely remain a therapeutic cornerstone in snakebite treatment for the immediate future, our *de novo* designed binders could potentially be used as fortifying agents to improve the efficacy of antivenoms; this would be particularly beneficial in the treatment of elapid envenomings, where low-molecular mass toxins with limited immunogenicity, but high medical importance, dominate the toxic effects of the venoms (and therefore must be neutralized)^59^. Beyond fortification, generative binder design could be used to generate neutralizing proteins against other medically relevant toxins, thereby expediting the discovery of antivenoms with broader species coverage. More generally, as *in silico* protein design is less resource intensive than traditional antibody development, our approach could aid in the democratization of drug design and discovery, enabling researchers residing in low and middle-income countries to better contribute to the development of effective treatments for snakebite envenoming and other neglected tropical diseases.

## METHODS

### Cytotoxin consensus sequence design

Amino acid sequences for cytotoxins were collected from the UniProt website using family:”snake three-finger toxin family Short-chain subfamily Type IA cytotoxin sub-subfamily” as a query. The resultant 86 unique CTX sequences thereafter underwent multiple sequence alignment (MSA) in Clustal Omega^1^. Using these alignments a consensus sequence was designed to represent the most common amino acids at each position across the aligned sequences. In this process, each column of the sequence alignment was analyzed to select the most frequent amino acid. In scenarios where no single amino acid dominated, a consensus symbol was used to represent a group of similar amino acids based on properties like charge or hydrophobicity. This approach allowed for the representation of conserved biochemical properties rather than specific amino acid identities at positions with high variability.

### Secondary structure and block adjacency tensors

In order to generate desired binder-target β-strand pairing interactions using RFdiffusion, fold-conditioning tensors describing single binder β-strands interacting with target β-strands in a matrix format were supplied to RFdiffusion at inference. This information is supplied via two tensors: a [L,4] secondary one-hot tensor (0=α-helix, 1=β-strand, 2=loop, 3=masked secondary structure identity) to indicate the secondary structure classification of each residue in the binder-target complex, and an [L,L,3] adjacency one-hot tensor (0=non-adjacent, 1=adjacent, 2=masked adjacency) to indicate interacting partner residues for each residue in the binder-target complex. For the design of the binders described here, the secondary structure tensor indicated an entirely masked binder structure with the exception of binder residues set to β-strand identities, while the adjacency tensor indicated a masked adjacency between binder-target residues with the exception of the pre-defined strand residues being adjacent to the defined target strand residues.

### *De novo* 3FTX binder design using RFdiffusion

The crystal structures of ScNtx (PDB ID: 7Z14) and α-cobratoxin (PDB ID: 1YI5) served as input for RFdiffusion. In the case of the consensus cytotoxin, its AF2 model was utilized. A total of approximately two thousand diffused designs were generated for each target, employing secondary structure and block adjacency tensors in the RFdiffusion model. The resulting backbone libraries underwent sequence design using ProteinMPNN, followed by FastRelax and AF2 + initial guess^2^. The resulting libraries were filtered based on AF2 PAE <10, pLDDT > 80, and Rosetta ddg < −40.

### Partial diffusion to optimize binders

The AF2 models of the highest-affinity designs for each toxin target were used as inputs to partial diffusion. The models were subjected to 10 and 20 noising timesteps out of a total of 50 timesteps in the noising schedule, and subsequently denoised (“diffuser.partial_T” input values of 10 and 20). Approximately two thousand partially diffused designs were generated for each target. The resulting library of backbones were sequence designed using ProteinMPNN after Rosetta FastRelax, followed by AF2+initial guess^2^. The resulting libraries were filtered based on AF2 PAE <10, pLDDT > 80, and Rosetta ddg < −40.

### Recombinant expression of ScNtx

ScNtx was recombinantly expressed from the methylotrophic yeast *Komagataella phaffii* (formerly known as *Pichia pastoris*). The ScNtx sequence was codon-optimized for expression in yeast and included a N-terminal His_6_ tag, followed by a Biotin Acceptor Peptide, and a Tobacco Etch Virus (TEV) proteolytic site. The expression was performed as previously described^3^. The culture media was dialyzed overnight against a wash buffer (50 mM Sodium phosphate buffer, pH 8.0, 20 mM imidazole). Purification was carried out using an NGC^™^ chromatography system (Bio-Rad) with a 5 mL IMAC Nickel column (Bio-Rad). After loading, the column was washed with 5 column volumes of wash buffer to remove non-specifically bound proteins. The protein was then eluted using a gradient of 250 mM imidazole over 10 column volumes. Fractions with high absorbance at 280 nm were pooled, dialyzed against 50 mM Sodium phosphate buffer, pH 8.0. Purity was assessed on SDS-PAGE to confirm the size. The protein solution was aliquoted, and stored at −20°C for further use.

### Toxins

α-cobratoxin (L8114) was obtained from Latoxan (Portes lés Valence, France). Cytotoxin from *Naja pallida* was obtained from Sigma-Aldrich (217503).

### Venoms

Whole venoms for initial neutralization screening from *Naja nigricollis* (CV01089563VEN) and *Naja pallida* (CV01089566VEN) were obtained in lyophilized form from Amerigo Scientific. Catalog numbers are provided in parentheses.

For *in vitro* neutralization experiments in human keratinocytes, whole venoms from *Naja nigricollis* (L1327), *Naja nigricincta* (L1368), *Naja mossambica* (L1376), *Naja nubiae* (L1342), *Naja katiensis* (L1317), *Naja ashei* (L1375), and *Naja pallida* (L1321) were purchased in lyophilized form from Latoxan (Portes lés Valence, France). Catalog numbers are provided in parentheses.

For the anti-cytotoxin *in vivo s*tudy, *Naja nigricollis* venom was sourced from wild-caught Tanzanian specimens housed in the herpetarium of the Liverpool School of Tropical Medicine.

### Gene construction of 3FTX binders

The designed protein sequences were optimized for expression in *E. coli*. Linear DNA fragments (eBlocks, Integrated DNA Technologies) encoding the design sequences contained overhangs suitable to cloning into the pETcon3 vector for yeast display (deposited in Addgene as #45121) and LM627 vector for protein expression (Addgene #191551) via Golden Gate cloning.

### Yeast display screening

For the yeast transformation, 50–60 ng of pETcon3, digested with NdeI and XhoI restriction enzymes, and 100 ng of the insert (eBlocks, Integrated DNA Technologies) were transformed into *S. cerevisiae* EBY100 following the protocol in^4^. EBY100 cultures were cultivated in C-Trp-Ura medium with 2% (w/v) glucose (CTUG). To induce expression, yeast cells initially grown in CTUG were transferred to SGCAA medium with 0.2% (w/v) glucose and induced at 30 °C for 16–24 h. After induction, cells were washed with PBSF (PBS with 1% (w/v) BSA) and labeled for 40 minutes with biotinylated toxin targets at room temperature using the without-avidity labeling condition^4^. Subsequently, cells were washed, resuspended in PBSF, and individually sorted based on each unique design using a 96-well compatible autosampler in the Attune NxT Flow Cytometer (Thermo Fisher Scientific).

### Protein expression and purification in *E. coli* for 3FTX binders

Protein expression was conducted in 50 mL of Studier autoinduction media supplemented with kanamycin, and cultures were grown overnight at 37°C. Cells were harvested by centrifugation at 4,000 × g for 10 min and resuspended in lysis buffer (100 mM Tris-HCl, 200 mM NaCl, 50 mM imidazole) supplemented with Pierce^™^ Protease Inhibitor Tablets (EDTA-free). Cell lysis was achieved by sonication using a Qsonica Q500 instrument with a 4-pronged horn for 2:30 min ON total, at an amplitude of 80%. Soluble fractions were clarified by centrifugation at 14,000 × g for 40 minutes and subsequently purified by affinity chromatography using Ni-NTA resin (Qiagen) on a vacuum manifold. Washes were performed using Low-salt buffer (20 mM Tris-HCl, 200 mM NaCl, 50 mM imidazole) and High-salt buffer (20 mM Tris-HCl, 1000 mM NaCl, 50 mM imidazole) before elution with Elution buffer (20 mM Tris-HCl, 200 mM NaCl, 500 mM imidazole). Eluted protein samples were filtered and injected into an autosampler-equipped Akta pure system on a Superdex S75 Increase 10/300 GL column at room temperature, using SEC running buffer (20 mM Tris-HCl, 100 mM NaCl, pH 8). Monodisperse peak fractions were pooled, concentrated using Spin filters (3 kDa molecular weight cutoff, Amicon, Millipore Sigma), and stored at 4°C before downstream characterizations. Protein concentrations were determined by absorbance at 280 nm using a NanoDrop spectrophotometer (Thermo Scientific) using the molecular weights and extinction coefficients obtained from their amino acid sequences using the ProtParam tool.

### Bio-layer Inferometry (BLI) Binding Experiments

BLI experiments were performed on an Octet Red96 (ForteBio) instrument, with streptavidin coated tips (Sartorius Item no. 18–5019). Buffer comprised 1X HBS-EP+ buffer (Cytiva BR100669) supplemented with 0.1% w/v bovine serum albumin. Tips were pre-incubated in the buffer for at least 10 minutes before use. Tips were then sequentially incubated in biotinylated toxin target, buffer, designed binder, and buffer.

### Affinity measurements by surface plasmon resonance (SPR)

SPR experiments were conducted using a Biacore^™^ 8K instrument (Cytiva) and analyzed with the accompanying evaluation software. Biotinylated α-cobratoxin was immobilized on a streptavidin sensor chip (Cytiva). For ScNtx and *Naja pallida’s* cytotoxin, immobilization involved the activation of carboxymethyl groups on a dextran-coated chip through reaction with N-hydroxysuccinimide. The ligands were then covalently bonded to the chip surface via amide linkages, and excess activated carboxyls were blocked with ethanolamine (doi: 10.1007/978-1-59745-523-7_20). Increasing concentrations of protein binders were flown over the chip in 1X HBS-EP+ buffer (Cytiva BR100669).

### Circular dichroism (CD)

Secondary structure content was evaluated by CD in a Jasco J-1500 CD spectrometer coupled to a Peltier system (EXOS) for temperature control. The experiments were performed on quartz cells with an optical path of 0.1 cm, covering a wavelength range from 200–260 nm. CD signal is reported as molar ellipticity [θ]. The thermal unfolding experiments were followed by a change in the ellipticity signal at 222 nm as a function of temperature. Proteins were denatured by heating the proteins at 1°C/min from 20 to 95°C.

### Crystallization and Structure Determination

Crystallization experiments for the binder complex were conducted using the sitting drop vapor diffusion method. Crystallization trials were setup in 200 nL drops using 96-well format by Mosquito LCP from SPT Labtech. Crystals drops were imaged using the UVEX crystal plate hotel system by JANSi. Diffraction quality crystals for LNG binder-complex appeared in 1.5 M Ammonium Sulfate and 25% (v/v) glycerol in 2 weeks. Diffraction quality crystals for SHRT binder appeared in 0.08 M Sodium acetate trihydrate pH 4.6, 1.6 M Ammonium sulfate and 20% (v/v) glycerol. For CYTX_B10-complex diffraction quality crystals appeared in 0.1 M MES pH 6, 0.01 M Zinc chloride, 20% (w/v) PEG 6000 and 10 % (v/v) Ethylene glycol. Crystals were flash-cooled in liquid nitrogen before shipping to the synchrotron for diffraction experiment.

Diffraction data were collected at the NSLS2 beamline AMX (17-ID-1). X-ray intensities and data reduction were evaluated and integrated by XDS^5^ and merged/scaled by Pointless/Aimless in the CCP4i2 program suite^6^. Structure determined by molecular replacement using a designed model using Phaser^7^. Following molecular replacement model was improved and refined by Phenix^8^. Model building was performed by COOT^9^ in between refinement cycles. Final model was evaluated by MolProbity^10^. Data collection and refinement statistics were reported in the Supplementary Table X. Final atomic coordinates, mmCIF and structure factors were deposited in the Protein Data Bank (PDB) with accession codes 9BK5, 9BK6 and 9BK7.

### *In vitro* neutralization using electrophysiology

Human-derived Rhabdomyosarcoma RD cells (American Type Culture Collection, ATCC), endogenously expressing the muscle-type nAChR were used for electrophysiology experiments^11^. Planar whole-cell patch-clamp recordings were conducted on a QUBE automated electrophysiology platform (Sophion Bioscience), with 384-channel patch chips (patch hole resistance 2.00 ± 0.02 MΩ), following the protocol detailed in^11^. Protein binders were preincubated with approximately 1 IC_80_ of α-cobratoxin or ScNtx at various toxin to binder molar ratios (1:1, 1:3, 1:9, and 1:27) and then added to the cells. The toxin’s ability to inhibit an acetylcholine (ACh, 70 μM) response, in the presence or absence of binders was normalized to the full ACh response and averaged within each group (n=16), and represented in a non-cumulative concentration-response plot. Data analysis was performed using Sophion Analyzer v6.6.70 (Sophion Bioscience) and GraphPad Prism v10.1.1 (GraphPad Software).Data analysis was performed using Sophion Analyzer v6.6.70 (Sophion Bioscience) and GraphPad Prism v10.1.1 (GraphPad Software).

### Initial neutralization screening of whole venoms using cell viability assay

HEK293T cells were cultured in DMEM (Dulbecco’s Modified Eagle Medium, Gibco) medium with 10% fetal bovine serum at 37 °C and 5% and CO_2_. Cells were subjected to commercial whole venoms from *Naja pallida* (34 μg/mL) and *Naja nigricollis* (42 μg/mL) either in the absence or presence of 1:1 or 5:1 molar ratio of toxin:binder. Buffer and binder-only controls were run in parallel and all samples were pre-incubated for 30 minutes at room temperature before addition to the HEK293T cells. To determine the percentage of viable cells, the RealTime-Glo^™^ MT Cell Viability Assay (Promega, Madison, WI, USA) was performed according to the manufacturer’s protocol. Experiments were performed in triplicates, and results were expressed as mean ± SD.

### *In vitro* neutralization of whole venoms using cell viability assay

N/TERT immortalized keratinocytes were cultured as previously described^12^. After determining the IC_50_ for seven venoms of Afronaja snakes, N/TERT cells were subjected to 2X the IC_50_ of each venom either in the absence or presence of a 1:5 molar ratio of venom:binder. Buffer and binder-only controls were run in parallel and all samples were pre-incubated (30 min at 37 °C) before addition to the N/TERT cells. To determine the percentage of viable cells, the CellTiter-Glo luminescent cell viability assay (Promega, Madison, WI, USA) was performed according to the manufacturer’s protocol. Experiments were performed in triplicates, and results were expressed as mean ± SD.

### LD_50_ determinations for α-neurotoxins

All assays used male NSA mice (20–30 g) and all doses were mass adjusted. Toxins assayed were α-cobratoxin (7820 Da, from *Naja kaouthia* venom obtained from Latoxan SAS, France) and short-chain neurotoxin ScNtx (8944 Da, recombinantly expressed). Toxins were solubilized in PBS at 1.0 mg/mL and then diluted in PBS as needed. For toxin LD_50_ determinations, five doses with 3 mice/dose were used, and a 100 μL bolus was injected IP in the right lower abdominal region; controls received only PBS. Injected mice were observed for the first 2 hours and then again at 24 hours; LD_50_ values were calculated using the “Quest Graph^™^ LD_50_ Calculator”^13^ (AAT Bioquest, Inc.; https://www.aatbio.com/tools/ld50-calculator).

### *In vivo* neurotoxicity protein binder protection assays

Toxins were used at 3X the LD_50_ (α-cobratoxin– 0.294 μg/g mouse; ScNtx – 0.261 μg/g mouse). In pre-incubation experiments, toxins were individually combined with a 10-fold molar excess of each protein binder (designed to bind each toxin specifically) in PBS and incubated at room temperature for 30 minutes. Mice (groups of 5) were injected with the binder:toxin protein as indicated above, observed for 2 hours and then again at 24 hours. For rescue-type experiments, toxins were IP administered (3X LD_50_) either 15 or 30 minutes before corresponding binder administration, either at 10- or 5-fold molar excess. Protection from toxin effects was scored as 24 hour percent mortality.

### *In vivo* dermonecrosis protein binder protection assays

Animal experiments were conducted under protocols approved by the Animal Welfare and Ethical Review Boards of the Liverpool School of Tropical Medicine and the University of Liverpool, and under project licence P58464F90 approved by the UK Home Office in accordance with the UK Animal (Scientific Procedures) Act 1986.

CD1 male mice (Charles River, 18–20 g) were acclimated for one week before experimentation in specific pathogen-free conditions. Holding room conditions were 23°C with 45–65% humidity and 12/12 hour light cycles (350 lux). Mice were housed in Techniplast GM500 cages (floor area 501 cm2) containing 120 g Lignocell wood fibre bedding (JRS, Germany), Z-nest biodegradable paper-based material for nesting and environmental enrichment (red house, clear polycarbonate tunnel and loft). Mice had *ad lib* access to irradiated PicoLab food (Lab Diet, USA) and reverse osmosis water in an automatic water system. Animals were split into cages (experimental units) upon arrival and no further randomisation was performed.

All mice were pre-treated with 5 mg/kg morphine (injected subcutaneously) before receiving intradermal injections in a 100 μL volume into the ventral abdominal region (rear side flank region). A venom-only control group of 5 mice received 63 μg of *Naja nigricollis* (Tanzania) venom (dissolved in PBS). For protection assays, crude venom was pre-incubated (30 min at 37 °C) with varying cytotoxin:binder ratios of 1:1, 1:2.5 and 1:5 prior to injection (n=3) (ratios estimated from the proportion of cytotoxin in the venom). Prior to this, a control group (N=3) received injections of cytotoxin binder alone (278 μM, equivalent to the 1:5 cytotoxin:binder dose) to check tolerance of the cytotoxin binder. For sample size, N of 3 was used for groups receiving cytotoxin binder as this was a pilot experiment. N of 5 was used for the venom-only control group due to variation in lesion size, and this is the size recommended by WHO. In total 17 mice were used. No inclusion or exclusion criteria were used during the experiment, and all data points were used in the analysis. No strategy was used to control for confounders. All experimenters were aware of the group allocation during the experiment and analysis.

After 72 hours, mice were euthanized by rising concentrations of CO_2_ and the lesions were excised. The outcome measured was the lesion size. Photographs of lesions were taken using a digital camera immediately after excision and the severity and size of dermonecrotic lesions was determined using VIDAL^14^.

## Figures and Tables

**Table 1.** Data collection and refinement statistics.

	LNG binder (holo) (PDB ID: 9BK5)	B10_CYTX binder (holo) (PDB ID: 9BK6)	SHRT_binder (apo) (PDB ID: 9BK7)
Resolution range	34.06 – 2.68 (2.85 – 2.68)	33.17 – 2.00 (2.05 – 2.00)	32.17 – 2.58 (2.84 – 2.58)
Space group	I 4_1_ 2 2	P 2_1_ 2_1_ 2_1_	I 4_1_ 2 2
Unit cell	77.79, 77.79, 173.52; 90, 90, 90	34.56, 63.66, 77.72; 90, 90, 90	75.33, 75.33, 108.34; 90, 90, 90
Unique reflections	9109 (1483)	12102 (864)	5179 (1262)
Multiplicity	24.3 (25.8)	6.4 (6.2)	24.6 (25.2)
Completeness (%)	99.86 (99.53)	99.7 (99.7)	99.83 (99.92)
Mean I/sigma(I)	17.12 (1.08)	10.4 (2.6)	13.30 (4.13)
Wilson B-factor	82.46	33.64	60.21
R-merge	0.105 (3.172)	0.088 (0.599)	0.246 (1.069)
R-pim	0.022 (0.631)	0.041 (0.281)	0.051 (0.215)
CC*	0.999 (0.522)	0.994 (0.929)	0.999 (0.982)
Reflections used in refinement	7836 (1264)	12047 (2928)	5179 (1262)
R-work	0.2387 (0.3049)	0.2496 (0.3301)	0.1970 (0.2954)
R-free	0.2681 (0.3349)	0.2850 (0.4167)	0.2235 (0.3560)
Number of non-hydrogen atoms	1118	1298	739
macromolecules	1118	1241	739
solvent	0	57	0
Protein residues	146	161	102
RMS(bonds)	0.003	0.002	0.004
RMS(angles)	0.55	0.45	0.62
Ramachandran favored (%)	92.25	96.82	97.00
Ramachandran allowed (%)	7.75	2.55	3.00
Ramachandran outliers (%)	0.0	0.64	0.00
Average B-factor	101.38	48.62	67.50
macromolecules	101.38	48.61	67.50
solvent	n/a	48.81	n/a

The highest-resolution shell are shown in parentheses.
